# Study of Reactive Melt Processing Behavior of Externally Plasticized Cellulose Acetate in Presence of Isocyanate

**DOI:** 10.3390/ma7127752

**Published:** 2014-12-04

**Authors:** Rafael Erdmann, Stephan Kabasci, Joanna Kurek, Stefan Zepnik

**Affiliations:** 1 Fraunhofer Institute for Environmental, Safety, and Energy Technology (UMSICHT), Osterfelder Straße 3, 46047 Oberhausen, Germany; E-Mails: rafael.erdmann@umsicht.fraunhofer.de (R.E.); stephan.kabasci@umsicht.fraunhofer.de (S.K.); joanna.kurek@umsicht.fraunhofer.de (J.K.); 2 Center of Engineering Sciences, Polymer Technology, Martin Luther University Halle-Wittenberg, 06099 Halle, Germany

**Keywords:** reactive melt processing, crosslinking, cellulose acetate, plasticizer, rheology, thermal properties

## Abstract

Two types of externally plasticized cellulose acetate (CA) were chemically modified using 4,4'-methylene diphenyl diisocyanate (MDI) as crosslinking agent. Crosslinking was performed in the molten state by means of melt mixing in an internal mixer. The viscoelastic properties of the non-crosslinked, externally plasticized CA show typical temperature dependence, similar to conventional thermoplastics. A strong increase in storage modulus is observed with increasing crosslink density indicating that the crosslinked compounds exhibit predominately elastic response. The complex viscosity also increases considerably with increasing crosslink density and does not reach the typical Newtonian plateau at low radial frequencies any more. The viscoelastic properties correlate well with the data recorded online during reactive melt processing in the internal mixer. In comparison to the non-crosslinked CA, the crosslinked compounds show higher glass transition temperature, higher VICAT softening temperatures, improved thermal stability and lower plasticizer evaporation at evaluated temperatures.

## 1. Introduction

Cellulose acetate (CA) was first invented by Schützenberger [1] in 1865 and is one of the oldest bio-based polymers in the world. It is produced from cellulose via esterification. CA is non-toxic, antiallergenic, and has excellent optical and dielectrical properties. In comparison to conventional thermoplastics such as polystyrene (PS) or polyolefins, CA is principally not melt processible without further modification. This is due to the narrow temperature window between melting and decomposition [2]. To this day, the addition of low molecular weight plasticizers is the predominant modification to obtain thermoplastic CA [2,3,4,5]. However, external plasticization has several drawbacks such as evaporation of plasticizer during melt processing at evaluated temperatures or leaching of plasticizer accompanied with property loss during service life.

To overcome these disadvantages and to improve the long-term properties of CA (e.g., light stability, thermal stability and/or dimensional stability), reactive melt processing is a promising approach. Partially substituted CA, which is mainly used for thermoplastics, is basically suitable for chemical modification (e.g., grafting or crosslinking) since it contains non-substituted free hydroxyl groups (OH groups). However, reactive melt modification of such partially substituted CA is difficult due to its high processing temperature and its high degree of substitution *DS* of 2.5 [6,7,8]. In addition, steric hindrances caused by the complex ring structure of the CA chains as well as internal hydrogen bonds between the non-substituted free OH groups lead to limited accessibility of these OH groups for reactions. Consequently, lots of studies describe the chemical modification of CA in solution [9,10,11,12,13,14,15,16]. During melt processing, mainly graft reactions onto CA have been studied [8,17,18,19,20,21,22]. Often, external plasticizers were added to improve processing and homogenization and to facilitate the grafting reaction in the molten state due to better access to the functional groups. Nevertheless, in most cases long reaction times and large quantities of catalysts and monomers are necessary for sufficiently high grafting yields [17,18,19,20,21,22]. Thus, most of the processing conditions used in these studies are not viable for typical compounding processes prohibiting economic feasibility of the processes. Beside grafting, partial crosslinking during melt processing could be another approach to achieve better stability and improved long-term properties of CA compounds. However, no detailed information is available concerning partial crosslinking of CA during melt processing. The aim of this paper is to present recent results of reactive melt processing of externally plasticized CA in presence of a crosslinking agent under residence times typical for compounding processes.

## 2. Experimental Section

### 2.1. Materials

Cellulose acetate was obtained from FKuR Kunststoff GmbH (Willich, Germany) in powder form with a mean particle diameter *d*_50_ of 200 µm. The *DS* is 2.5 and the combined acetic acid content is between 54.5% and 55.6%. The CA used has a molecular weight *M*_w_ of about 185,600 g·mol^−1^ and a polydispersity *PD* of 3.5. The density ρ is 1.33 g·cm^−3^ and the refractive index *n_D_* is 1.47. Two different plasticizers, namely glycerol triacetate (GTA) and triethyl citrate (TEC), were used for improving melt processing and flowability of CA. The molar mass *M*_n_, the density ρ, the refractive index *n_D_*, and the boiling point *bp* of these plasticizers are listed in Table 1.

**Table 1:** Main characteristics of glycerol triacetate and triethyl citrate.

Plasticizer	*M*_n_ (g mol^−1^)	*Ρ* (g cm^−3^)	*n*_D_ (–)	*bp* (1013 mbar) (°C)
GTA	218.21	1.16	1.43	258
TEC	276.28	1.14	1.44	294

**Figure 1:**
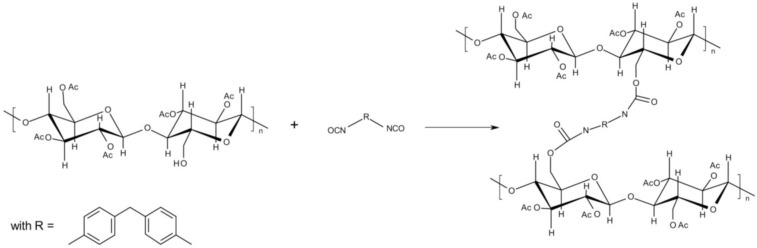
Scheme of isocyanate crosslinking of cellulose acetate.

4,4'-Methylene diphenyl diisocyanate (MDI) was used as crosslinking agent. MDI was obtained from BASF Polyurethanes GmbH (Lemförde, Germany) as solid masterbatch. The matrix of the MDI masterbatch is a thermoplastic polyester-polyurethane. The total amount of free, non-reacted MDI in the masterbatch is 25 wt% and the density is 1.22 g cm^−3^. Figure 1 shows the crosslinking reaction between the isocyanate groups and the free OH groups of the partially substituted CA with a *DS* of 2.5.

### 2.2. Reactive Melt Processing Conditions and Online Measurements

**Figure 2:**
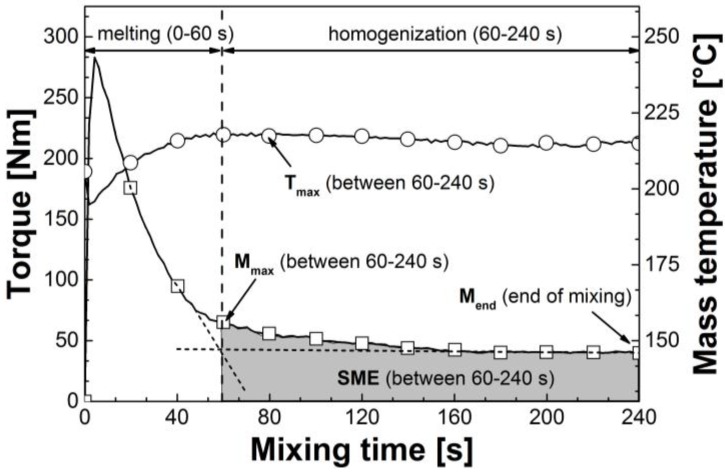
Typical plastogram of externally plasticized CA (CA/GTA 85/15).

The plasticizer concentration was kept constant at 15 wt% for all test runs. Different concentrations of the MDI masterbatch were studied, namely 0.75, 2.5, 5, and 7.5 wt%. This corresponds with a total non-reacted MDI content in the compounds of 0.19, 0.63, 1.25, and 1.88 wt%, respectively. The reactive melt processing was done using an internal mixer from Brabender (Duisburg, Germany). All tests were performed in nitrogen atmosphere at a chamber temperature of 210 °C with a rotational speed of the blades of 60 min^−1^. To evaluate the reactive melt processing of externally plasticized CA under typical compounding conditions, maximum mixing time was fixed at 4 min. At first, CA was dried for 6 h at 100 °C and then dry blended with the selected plasticizer and the MDI masterbatch at room temperature in a powder mixer. After that, the dry blends were fed into the preheated chamber of the internal mixer. Mass temperature *T* and torque *M* were measured and recorded online during melt mixing. Figure 2 shows a typical plastogram, *i.e.*, the plot of temperature and torque against the mixing time, of externally plasticized CA without crosslinking agent. The first stage of mixing is mainly attributed to melting of the dry blend. The sharp peak at the beginning of mixing represents complete filling of the chamber with the solid dry blend. After approx. 60 s of mixing, a change in the slope of the torque curve can be observed. At this point, the dry blend is almost completely molten and the main homogenization of the mixture starts.

The reactive melt processing behavior of externally plasticized CA was investigated using characteristic melt mixing data during the homogenization step between 60 and 240 s of the mixing process. These data include the maximum torque *M*_max_, the average torque, the maximum mass temperature *T*_max_ as well as the final torque at the end of mixing *M*_end_. In addition, the specific mechanical energy input *SME* of the homogenization step was calculated from the torque-time graphs according to Equation (1) [23]:

(1) $$SME=\frac{\omega }{m}\cdot \int_{60}^{240}C(t)dt\;(J\;g^{-1})$$

where ω is the rotor speed, *m* is the sample mass, *C*(*t*) is the torque at time *t* = 60 s to *t* = 240 s (end of mixing).

### 2.3. Viscosimetry and Further Investigations of the Products

The influence of crosslinking agent on the viscoelastic properties of externally plasticized CA was studied in terms of storage (elastic) modulus *G'*, loss (viscous) modulus *G''*, complex modulus G*, and complex viscosity η*. The measurements were conducted in plate-plate modus at 210 °C, 220 °C, and 230 °C. The linear elastic region (LVR) was measured for each temperature by using a shear stress amplitude sweep ranging from 0.1 to 10,000 Pa at two constant frequencies of 0.01 and 100 Hz. Then, the viscoelastic properties of the samples were determined using frequency sweeps ranging from 0.01 to 100 Hz at constant shear stress of 500 Pa for all temperatures.

To verify the crosslinking success during melt mixing, solubility tests were conducted in typical solvents for CA, namely acetone, dimethylformamide (DMF), dimethyl sulfoxide (DMSO), and tetrahydrofuran (THF). For this, 10 mg of each compound was dissolved in 10 mL solvent under permanent stirring at room temperature for 24 h. The solubility was evaluated qualitatively. The crosslinking yield *X*_y_ was measured for quantitative assessment according to Equation (2) [24]:

(2) $$X_{y}=\left(\frac{P_{s}}{P_{i}}\right)\cdot 100\%\;(\%)$$

where *P_s_* is the weight of the dried filter cake (undissolved gel) and *P_i_* is the weight of the dried compound before swelling. A sample mass of 30 mg was dissolved in 30 mL acetone at 60 °C under permanent stirring, nitrogen atmosphere, and reflux conditions. After 24 h, the gel was separated from the solution by filtration and was dried for 48 h in a vacuum dryer at 60 °C and 15 mbar. The dried filter cake was then weighted and referred to the initial sample mass according to Equation (2). Each compound was measured three times and the average value was used as crosslinking yield *X*_y_. The reactive melt mixed compounds and the dried filter cake were further characterized by Fourier transform infrared spectroscopy (FTIR) (Vertex 70; Bruker Corporation, Billerica, MA, USA).

Thermal properties were investigated by means of differential scanning calorimetry (DSC) (DSC 204; Netzsch Gerätebau GmbH, Selb, Germany), VICAT softening temperature (VST) measurement (Vicat/HDT-Tester; Coesfeld GmbH & Co. KG, Dortmund, Germany), and thermogravimetry (TG) (F209 IRIS; Netzsch Gerätebau GmbH). DSC experiments were conducted from −50 to 250 °C with a heating and cooling rate of 10 K min^−1^ as well as an isotherm of 3 min at −50 °C and 250 °C. Characteristic thermal transitions were obtained from the second heating cycle. TG measurements were performed from 25 to 550 °C with a heating rate of 10 K min^−1^ in nitrogen atmosphere. VST was measured according to DIN EN ISO 306:2014-03 [25] with a load of 10 N and a heating rate of 50 K h^−1^. This method is also known as VST A50.

## 3. Results and Discussion

### 3.1. Reactive Melt Processing

**Figure 3:**
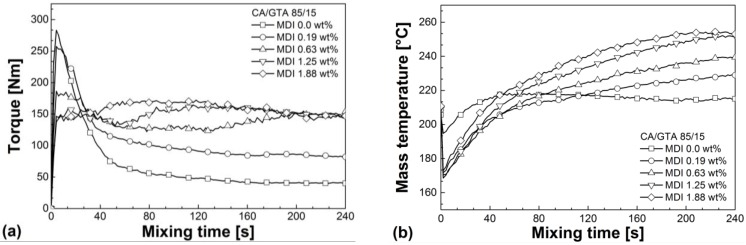
(**a**) Torque as a function of mixing time for cellulose acetate/glycerol triacetate/4,4'-methylene diphenyl diisocyanate (CA/GTA/MDI) in dependence of MDI content. (**b**) Mass temperature as a function of mixing time for CA/GTA/MDI in dependence of MDI content.

Figure 3 shows the influence of MDI on the reactive melt processing of CA/GTA (85/15). Similar graphs were obtained for CA/TEC (85/15). Without crosslinking agent, externally plasticized CA exhibits no significant change in torque and mass temperature during homogenization indicating no significant change in viscosity, e.g., due to chemical reactions or degradation. The addition of MDI leads to higher torque levels and a steady increase in mass temperatures. It can be assumed that the crosslinking reaction between the isocyanate and the free OH groups of CA results in the formation of chemically bonded network structures, which strongly increases the melt viscosity. This causes significant higher torque levels. The higher viscosity causes principally higher shear induced dissipation. In combination with the exothermal nature of the crosslinking reaction this leads to a significant increase in mass temperature over mixing time. However, especially for high MDI contents, even increasing torque values can be found in spite of the strong temperature rise that takes place in parallel.

The higher the MDI content, the earlier the crosslinking reaction starts. This is reflected by a shift of the starting point of the torque increase towards lower mixing times, as can be seen in Figure 3a. Contrary, the final torque *M*_end_ at the end of mixing seems to be independent from the crosslinking agent at higher MDI contents (0.63 wt% or higher).

**Figure 4:**
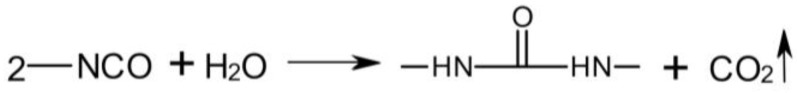
Scheme of isocyanate reaction with water.

As can be further seen from Figure 3a, at low MDI content (0.19 wt%) the increase of the torque level is fairly moderate indicating limited crosslinking reaction, which could be attributed to competitive side reactions taking place during melt mixing. Because CA is highly hydrophilic, it must be assumed that a certain amount of moisture is internally bonded between the free OH groups, despite that CA was carefully dried before melt mixing. In this case, the isocyanate will more readily react with the water instead of the free OH groups, as shown schematically in Figure 4. Gao *et al.* [26] studied the effect of moisture on the reaction of isocyanate with wood. They also report this preferred reaction of isocyanate with water in wood. One can suppose that at low MDI content, this competitive reaction between isocyanate and residual moisture in CA results in the consumption of most of the isocyanate and no noteworthy crosslinking reaction with the free OH groups of CA takes place. At sufficiently high MDI content, an excess of isocyanate is present in the melt and adequate crosslinking can occur.

**Table 2:** Characteristic melt mixing data during homogenization step (60–240 s) for CA/GTA/MDI and CA/TEC/MDI. avg. means average.

Mixtures	*M*_max_ (60–240 s) (Nm)	avg. *M* (60–240 s) (Nm)	*M*_end_ (Nm)	*T*_max_ (60–240 s) (°C)	*SME* (60–240 s) (J g^−1^)
CA/GTA 85/15					
+ MDI 0	65.3	46.1	39.9	218.4	173.6
+ MDI 0.19	109.3	90.0	81.9	229.3	339.0
+ MDI 0.63	154.6	139.2	148.6	239.5	514.6
+ MDI 1.25	164.4	154.0	147.5	252.3	580.8
+ MDI 1.88	172.1	159.3	147.1	254.4	600.6
CA/TEC 85/15					
+ MDI 0	105.6	87.2	70.5	224.7	308.6
+ MDI 0.19	111.8	89.9	83.2	228.9	338.3
+ MDI 0.63	116.0	104.5	107.0	233.6	393.8
+ MDI 1.25	151.6	131.5	142.1	243.6	477.3
+ MDI 1.88	175.6	158.6	159.1	248.0	597.7

Table 2 summarizes the reactive melt mixing data of the homogenization step for CA/GTA/MDI and CA/TEC/MDI. The data confirm the above mentioned results. Increasing MDI contents leads to stronger network structure formation with higher crosslink density. This increases the melt viscosity, which in turn causes higher torque values, higher *SME* values as well as higher dissipation induced mass temperatures. Cailloux *et al.* [27] also found a strong torque increase during reactive melt mixing of PLA using an epoxy-based chain extender. They also explain this with an increase in viscosity due to branching reaction and network structure formation. Furthermore, Klébert [19] as well as Klébert *et al.* [20] reported a similar strong increase in torque during melt grafting of CA with different caprolactones.

### 3.2. Solubility Tests

Solubility tests in typical CA solvents were conducted to verify the crosslinking success. Table 3 shows qualitatively and quantitatively the solubility of CA/GTA/MDI and CA/TEC/MDI. As can be seen, the crosslinking yield *X*_y_ increases steadily with increasing MDI content. However, a certain amount of crosslinking agent seems to be necessary to obtain noticeable crosslinks in CA during melt mixing as complete dissolution is observed at very low MDI content (0.19 wt%), irrespective of plasticizer type. This confirms the hypothesis made from the melt mixing data that at very low MDI content no substantial crosslinking took place due to the competitive reaction of the isocyanate with the residual moisture internally bonded in CA. At MDI content of 0.63 wt% or higher, the compounds exhibit swelling and only partial solubility, irrespective of plasticizer and solvent. The higher the MDI content, the higher the crosslinking yield and the lower the solubility. From the solubility tests, one can conclude that the reactive melt mixing of externally plasticized CA using MDI content of 0.63 wt% or higher was successful.

**Table 3:** Solubility characteristics and crosslinking yield (determined in acetone) with standard deviation in parenthesis for CA/GTA/MDI and CA/TEC/MDI.

Mixtures	Acetone	DMF	DMSO	THF	*X*_y_ in acetone [%]
CA/GTA 85/15					
+ MDI 0	+	+	+	+	0
+ MDI 0.19	+	+	+	+	0
+ MDI 0.63	−	−	−	−	41 (1.81)
+ MDI 1.25	−	−	−	−	52 (1.75)
+ MDI 1.88	−	−	−	−	56 (2.18)
CA/TEC 85/15					
+ MDI 0	+	+	+	+	0
+ MDI 0.19	+	+	+	+	0
+ MDI 0.63	−	−	−	−	38 (0.31)
+ MDI 1.25	−	−	−	−	45 (1.76)
+ MDI 1.88	−	−	−	−	49 (1.47)

**+** = soluble; **−** = (partial) insoluble.

**Figure 5:**
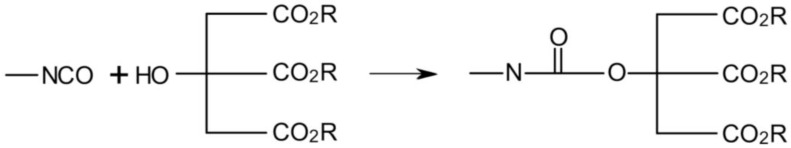
Scheme of isocyanate reaction with triethyl citrate.

From Table 3, it can further be seen that slightly higher values of *X*_y_ are observed for CA/GTA/MDI than for CA/TEC/MDI, which is also in good agreement with the melt mixing data (Section 3.1) and the rheological results (Section 3.3). Here, CA/GTA/MDI exhibits higher values for the torque, the *SME* as well as the viscoelastic properties. Despite of the hydroxyl group in TEC being a low-reactive tertiary one, a certain amount of MDI might react with that free OH group and not with the primary or secondary OH groups of the partially substituted CA. This is schematically shown in Figure 5. The higher mobility of TEC (lower molecular weight) without ring structures in the molecule results in a fairly good accessibility of the OH group of TEC, in comparison to the rigid CA chains with complex glucose ring structures and steric hindrances.

**Figure 6:**
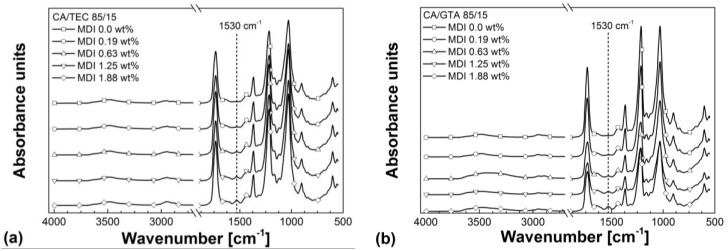

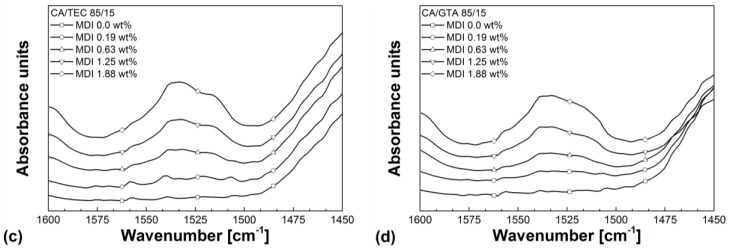
(**a**) Infrared spectra of CA/TEC/MDI in dependence of MDI content; (**b**) Infrared spectra of CA/GTA/MDI in dependence of MDI content; (**c**) Detailed view of the infrared spectra of CA/TEC/MDI; and (**d**) Detailed view of the infrared spectra of CA/GTA/MDI.

FTIR spectra in Figure 6 compare the non-crosslinked CA/GTA (85/15) and CA/TEC (85/15) with the crosslinked compounds. As can be seen, the isocyanate crosslinked compounds show a characteristic absorption band between 1500 and 1570 cm^−1^, which is neither present in the non-crosslinked CA/GTA (85/15) nor in the non-crosslinked CA/TEC (85/15). This absorption band might be referred to N–H deformation and C–N stretching vibration of the carbonyl-urethane group being present in the crosslinked compounds. Additionally, the intensity of this infrared absorption increases constantly with increasing crosslinking agent content. The characteristic absorption band of the carbonyl-urethane group for isocyanate crosslinked CA is also observed by other researchers [28,29]. Furthermore, Xing *et al.* [30] described the characteristic N–H deformation around 1530 cm^−1^ in gelatin hydrogels. However, additional analytical methods have to be used (e.g., solid-state nuclear magnetic resonance (NMR) spectroscopy) to obtain further information on the complex network structure of the crosslinked compounds and in case of CA/TEC (85/15) to elucidate whether a certain amount of MDI is grafted onto TEC or not.

### 3.3. Rheological Properties 

**Figure 7:**
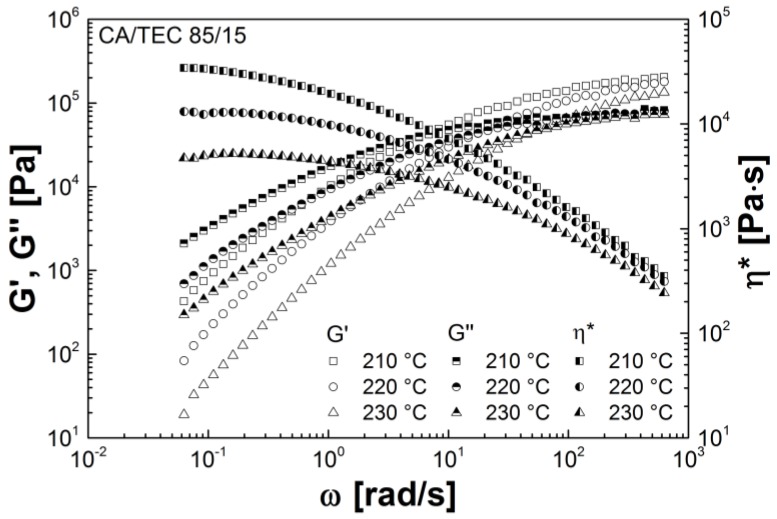
Storage modulus, loss modulus, and complex viscosity of CA/TEC (85/15) as a function of radial frequency dependent on temperature.

Figure 7 shows the storage modulus *G'*, the loss modulus *G''*, and the complex viscosity η* of CA/TEC (85/15) at 210 °C, 220 °C, and 230 °C. Similar curves are observed for CA/GTA (85/15). At higher radial frequencies, the externally plasticized CA shows typical shear thinning behavior, irrespective of plasticizer type and temperature. This is reflected by a steady decrease in complex viscosity with increasing radial frequency. Furthermore, storage modulus, loss modulus as well as complex viscosity decreases with increasing temperature. The intersection point of *G'* and *G''* shifts to higher radial frequencies with increasing temperature. At higher temperatures, mobility and flexibility of the CA chains is higher and disentanglement is easier. The relaxation time decreases and the intersection from viscous dominated behavior to elastic dominated behavior shifts towards higher radial frequencies. Overall, externally plasticized CA, which is not crosslinked, shows a rheological behavior typical for conventional thermoplastics.

Time-temperature superposition was used to obtain temperature-dependent master curves for the moduli and the complex viscosity. For this, a temperature shift factor *a_T_* was calculated according to Equation (3) [31]:

(3) $$a_{T}=\frac{\omega \left(T_{\text{ref}}\right)}{\omega \left(T_{i}\right)}\;(–)$$

where ω(*T*_ref_) is the radial frequency at the intersection of *G'* and *G''* at the reference temperature *T*_ref_, and ω(*T_i_*) is the radial frequency at the intersection of *G'* and *G''* at temperature *T_i_*.

**Figure 8:**
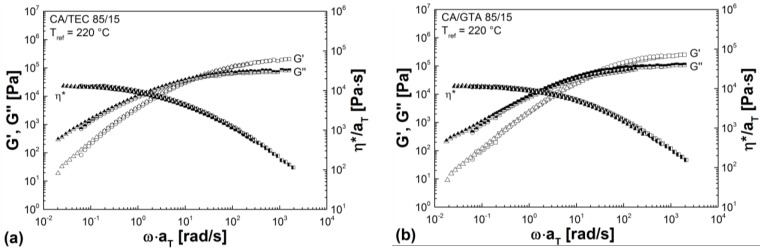
(**a**) Temperature-dependent master curves for non-crosslinked CA/TEC (85/15) as a function of radial frequency. (**b**) Temperature-dependent master curves for non-crosslinked CA/GTA (85/15) as function of radial frequency.

Figure 8 shows the temperature-dependent master curves for non-crosslinked CA/TEC (85/15) in comparison to non-crosslinked CA/GTA (85/15). As can be seen from Table 4 and Figure 8, slightly lower moduli (30% for *G'* and 27% for *G''*) and slightly lower complex viscosity (15% for η*) are observed in the low frequency region for CA/GTA (85/15) indicating stronger plasticization and dilution of the CA matrix by GTA than by TEC. This is in good agreement with the results from the melt mixing (Section 3.1) and the thermal properties (Section 3.4), where non-crosslinked CA/GTA (85/15) shows lower torque values, lower *SME*, and lower glass transition than non-crosslinked CA/TEC (85/15).

**Figure 9:**
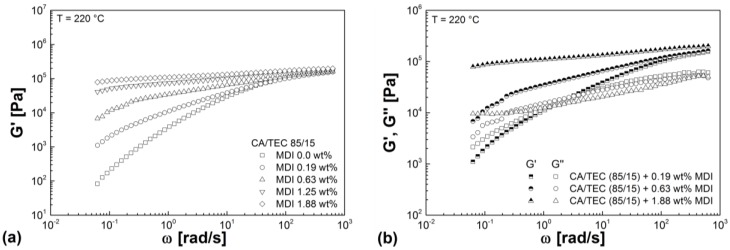
(**a**) Storage modulus of CA/TEC/MDI as a function of radial frequency in dependence of MDI content. (**b**) Storage modulus and loss modulus as a function of radial frequency for CA/TEC (85/15) with 0.19 wt%, 0.63 wt%, and 1.88 wt% MDI.

As can be seen from Figure 9a, a continuous increase in *G'* is observed with increasing MDI content. Similar results were obtained for CA/GTA/MDI. Chetty *et al.* [32] also reported a steady increase in the viscoelastic properties of poly(*N*-isopropylacrylamide-*co*-*N*,*N*'-methylene-bis-acrylamide) hydrogels with increasing crosslinking agent. The crosslinking reaction between the isocyanate and the free OH groups of CA leads to the formation of chemically bonded network structures, which strongly increases the stiffness (elastic modulus) in the low frequency range. The higher the crosslinking agent content, the higher the degree of crosslinking and thus the higher *G'* in the low frequency range. According to Winter and Mours [33], this means that the relaxation time strongly increases with increasing crosslink density and elastic response is predominant. A further indication is that at high MDI content (1.25 and 1.88 wt%), *G'* and *G''* exhibit extremely low frequency dependence and running in parallel without an intersection point. Wolff *et al.* [34] described also this parallelism of *G'* and *G''* for pre-crosslinked silicone resins. From Figure 9b and Table 4, it can be further seen that *G'* shows a stronger increase than *G''* with increasing MDI content and thus being significantly higher than *G''* in case of the highly crosslinked compounds. These effects have been reported by many researchers for different polymers [30,31,35,36] and shows that elastic response is predominant in highly crosslinked compounds.

**Figure 10:**
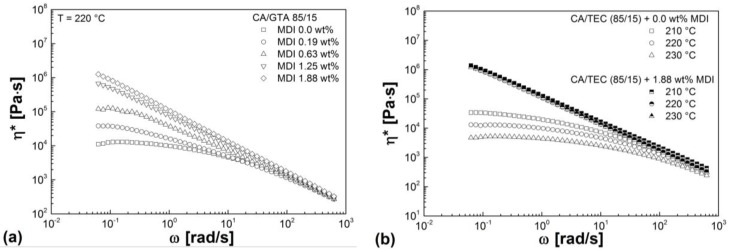
(**a**) Complex viscosity of CA/TEC/MDI as a function of radial frequency in dependence of MDI content. (**b**) Complex viscosity of CA/TEC (85/15) in comparison to CA/TEC (85/15) with 1.88 wt% MDI as a function of radial frequency in dependence of temperature.

The influence of crosslinking agent on the complex viscosity of CA/TEC (85/15) is shown in Figure 10a. As expected, increasing MDI content leads to a steady increase in complex viscosity due to increasing degree of crosslinking of the compound. Similar curves are obtained for CA/GTA/MDI. At high MDI content (1.25 and 1.88 wt%), no Newtonian plateau (zero viscosity) is reached due to the gel-like character of the crosslinked materials. Similar results can be found in literature, e.g., [31,32,33]. As can be seen from Figure 10b, the complex viscosity of highly crosslinked CA/TEC (85/15) (high MDI content) have lower dependency on temperature as compared to the non-crosslinked CA/TEC (85/15). The same results were achieved for CA/GTA/MDI.

For better comparison, Table 4 summarizes the viscoelastic melt properties of CA/GTA/MDI and CA/TEC/MDI at low frequency of 0.01 Hz and 220 °C dependent on MDI content. When MDI is added to CA/GTA (85/15), obviously higher values for the viscoelastic properties are obtained than for CA/TEC (85/15). Together with the melt mixing data (Section 3.1), this indicates stronger network structure formation (higher crosslink density) in the case of CA/GTA (85/15). The higher crosslinking yield *X*_y_ in comparison to CA/TEC/MDI confirms this hypothesis (Section 3.2). Regardless of the plasticizer type, for all compounds having MDI content of 0.63 wt% or higher *G'* is almost one order of magnitude higher than *G''* indicating predominant elastic response.

**Table 4:** Viscoelastic properties for CA/GTA/MDI and CA/TEC/MDI at 0.01 Hz and 220 °C.

Mixtures	*G*' (Pa)	*G*'' (Pa)	G* (Pa)	η* (Pa s)
CA/GTA 85/15				
+ MDI 0	5.7 × 10^1^	5.1 × 10^2^	5.1 × 10^2^	1.1 × 10^4^
+ MDI 0.19	1.4 × 10^4^	2.2 × 10^4^	2.6 × 10^4^	4.2 × 10^5^
+ MDI 0.63	2.0 × 10^5^	3.8 × 10^4^	2.1 × 10^5^	3.3 × 10^6^
+ MDI 1.25	3.9 × 10^5^	5.3 × 10^4^	3.9 × 10^5^	6.2 × 10^6^
+ MDI 1.88	4.8 × 10^5^	6.6 × 10^4^	4.9 × 10^5^	7.8 × 10^6^
CA/TEC 85/15				
+ MDI 0	8.3 × 10^1^	7.0 × 10^2^	7.0 × 10^2^	1.3 × 10^4^
+ MDI 0.19	1.1 × 10^3^	2.1 × 10^3^	2.4 × 10^3^	3.8 × 10^4^
+ MDI 0.63	6.8 × 10^3^	3.4 × 10^3^	7.6 × 10^3^	1.3 × 10^5^
+ MDI 1.25	4.1 × 10^4^	9.4 × 10^3^	4.2 × 10^4^	6.7 × 10^5^
+ MDI 1.88	7.9 × 10^4^	9.6 × 10^3^	7.9 × 10^4^	1.3 × 10^6^

**Figure 11:**
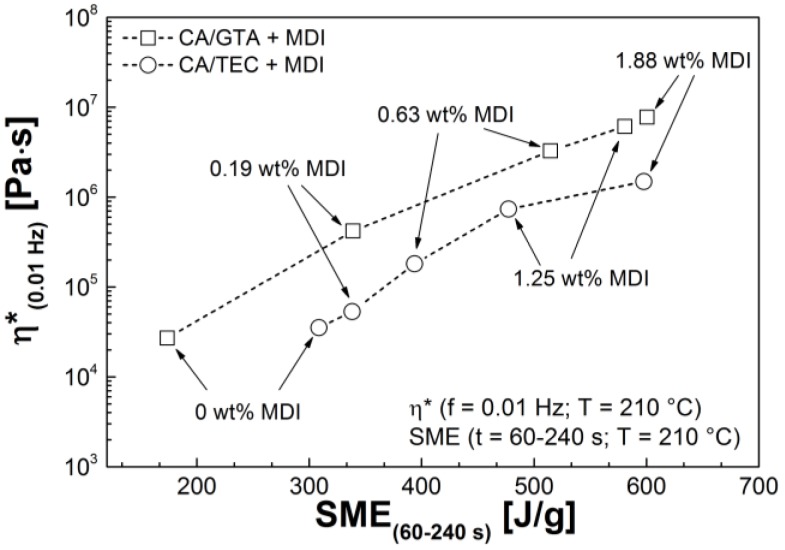
Complex viscosity at 0.01 Hz as a function of specific mechanical energy input for CA/GTA/MDI and CA/TEC/MDI.

Figure 11 shows the complex viscosity of CA/TEC/MDI and CA/GTA/MDI at 0.01 Hz and 210 °C plotted against the *SME* calculated from the torque-time graphs (Section 3.1). Both, CA/TEC/MDI and CA/GTA/MDI, show the same trend, irrespective of the plasticizer. The complex viscosity increases with increasing *SME*. The higher the crosslinking agent content, the higher the crosslink density, which in turn results in higher viscosity and *SME* values for melt processing. The non-crosslinked CA/GTA (85/15) shows lower complex viscosity and lower *SME* than non-crosslinked CA/TEC (85/15). However, when MDI is added, the complex viscosity as well as the *SME* is significantly higher for CA/GTA/MDI, which is in good agreement with the higher crosslinking yield *X*_y_ (Section 3.2).

### 3.4. Thermal Properties

**Figure 12:**
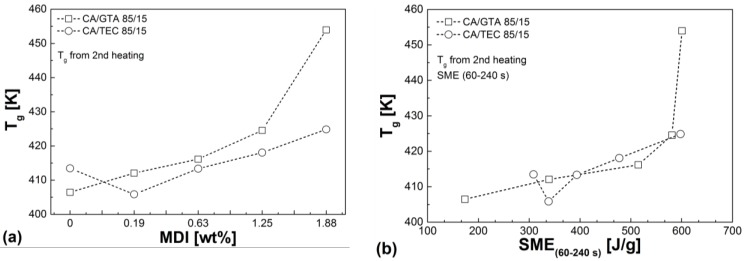
(**a**) Glass transition of CA/GTA (85/15) and CA/TEC (85/15) as a function of MDI content. (**b**) Glass transition of CA/GTA/MDI and CA/TEC/MDI as a function of calculated SME.

The glass transition temperature *T*_g_ is shown in Figure 12 as a function of the MDI content and plotted against the calculated *SME*. With increasing MDI content, *T*_g_ increases due to an increase in crosslink density. Crosslinking reduces the thermally activated number of chains and the chain mobility, and thus increases the glass transition temperature. This result is in good line with the results shown in the previous Section 3.1, Section 3.2 and Section 3.3 and is in good agreement with literature data, especially for epoxy systems [37,38,39,40]. The increase in *T*_g_ of the isocyanate crosslinked CA is accompanied by a steady increase in the VICAT softening temperature VST A50, as can be seen from Table 5. The values obtained for CA/GTA/MDI are principally higher than for CA/TEC/MDI, which is in good agreement with the previous results from Section 3.1, Section 3.2 and Section 3.3.

**Figure 13:**
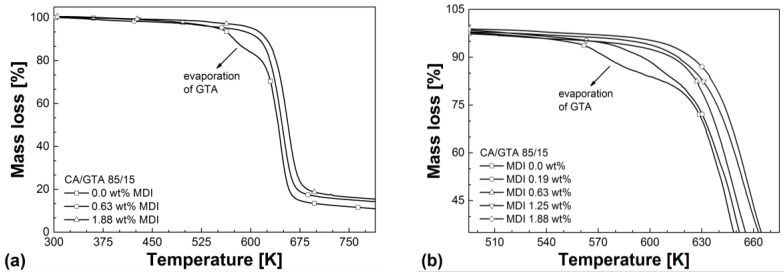
(**a**) Mass loss as a function of temperature for CA/GTA (85/15) in comparison to CA/GTA (85/15) with 0.63 wt% MDI and 1.88 wt%. (**b**) Mass loss as a function of temperature for CA/GTA/MDI in dependence of MDI content.

From Figure 13a, it can be seen that externally plasticized CA exhibits two-step degradation. The first degradation step correlates very well with the boiling point of the plasticizer (in this case: GTA). The second degradation step is related to the decomposition of CA. CA/TEC (85/15) showed a similar two-step degradation behavior. However, the onset decomposition temperature of the first step is slightly higher due to the higher boiling point of TEC.

Crosslinking of externally plasticized CA leads to an increase in thermal stability, as it is shown in Figure 13a,b. Ghatge *et al.* [13] also reported an improvement in thermal stability of crosslinked CA membranes. Reddy and Yang [41] as well as Vera-Graziano [24] found a similar improvement in thermal stability of citric acid crosslinked starch and crosslinked polydimethylsiloxane, respectively. The higher the crosslinking agent content the higher the degree of crosslinking and, consequently, the higher the thermal stability of CA. This trend is independent from the plasticizer investigated in this study, as can be seen from Table 5. Figure 13b shows that crosslinking of externally plasticized CA results in one-step degradation without early plasticizer loss. Therefore the decomposition behavior of the crosslinked compounds is similar to pure CA, which contains no plasticizer [2]. Increasing crosslink density reduces the free volume and chain mobility. This reduces the transport properties of the polymer matrix and consequently leads to lower plasticizer evaporation from the CA compound at evaluated temperatures. Ambrogi *et al.* [42] as well as Lakshmi and Jayakrishnan [43] also found a reduced plasticizer migration in crosslinked polyvinyl chloride (PVC). However, Ambrogi *et al.* [42] did not observe an improvement in thermal stability of crosslinked PVC. They attribute this to additional HCL, which is produced by the crosslinking agent isophoron diamine during degradation.

Table 5 summarizes the major thermal properties measured in this study. As described previously, glass transition temperature, VST A50, and decomposition temperature continuously increase with increasing crosslinking agent content. Thus, the crosslinked CA compounds show improved thermal stability and higher service temperatures in comparison to the non-crosslinked CA. One can also see from Table 5 that non-crosslinked CA/GTA (85/15) has a slightly lower glass transition temperature than non-crosslinked CA/TEC (85/15). This indicates further the stronger plasticization and dilution effect of GTA in comparison to TEC. The lower glass transition for CA/GTA (85/15) is very well in line with the results from Section 3.1 (lower torque, lower *SME*) and Section 3.3 (lower viscoelastic properties). However, when MDI is added and crosslinking took place, CA/GTA/MDI always shows higher glass transition, higher VST A50, and higher thermal stability than CA/TEC/MDI.

**Table 5:** Characteristic thermal properties of CA/GTA/MDI and CA/TEC/MDI.

Mixtures	*T*_g_ (2nd heating) (K)	*T*_d_ (50% mass loss) (K)	Residue (%)	VST A50 (K)
CA/GTA 85/15				
+ MDI 0	406.5	642	10.1	394.9
+ MDI 0.19	412.1	644	10.6	401.2
+ MDI 0.63	416.2	648	13.9	410.5
+ MDI 1.25	424.6	654	15.2	420.1
+ MDI 1.88	454.0	657	15.7	448.7
CA/TEC 85/15				
+ MDI 0	413.5	640	10.5	398.2
+ MDI 0.19	405.9	638	10.3	400.5
+ MDI 0.63	413.4	645	12.7	404.5
+ MDI 1.25	418,1	649	13.7	411,3
+ MDI 1.88	424,9	653	15.3	419,5

## 4. Conclusions

Crosslinking of externally plasticized cellulose acetate (CA) during melt processing has been studied. Externally plasticized CA was successfully crosslinked via melt mixing in sufficiently low reaction times using 4,4'-methylene diphenyl diisocyanate (MDI) as crosslinking agent. This can already be noticed in processing by a significant increase in torque, specific mechanical energy input and mass temperature due to increasing crosslink density. Solubility tests and FTIR spectroscopy verified the success of crosslinking. The highly crosslinked compounds do not dissolve in typical solvents for CA and the crosslinking yield increases with increasing isocyanate content. The viscoelastic properties as well as the complex viscosity increase with increasing crosslink density. The crosslinked CA compounds predominantly show elastic response and the complex viscosity does not reach a Newtonian plateau at low radial frequencies anymore. Storage modulus and loss modulus graphs are running in parallel without an intersection point. The crosslinking of externally plasticized CA reduces the temperature dependence of the viscoelastic properties. The crosslinked compounds showed noticeably higher glass transition temperature, higher VICAT softening temperature, and improved thermal stability. TG measurements revealed one-step degradation for the crosslinked CA, contrary to the non-crosslinked CA with a two-step degradation caused by plasticizer evaporation at elevated temperatures. However, the study also showed that a certain minimum amount of MDI is required to achieve sufficient crosslinking. At too low isocyanate content, the competitive reaction between the isocyanate and the residual moisture in CA overlies the reaction with the free OH groups of CA. The chemical structure of the plasticizer used also seems to have certain relevance during crosslinking as side reactions can occur, e. g. between the isocyanate and the OH group in TEC. 

From the results obtained in this study one can conclude that crosslinking of the CA matrix seems to be more efficient in case of CA/GTA than in case of CA/TEC. However, to verify this hypothesis additional characterization of the compounds is required. For this, NMR spectroscopy as well as TG measurements coupled with FTIR will be conducted to obtain further information on the network structure of the crosslinked CA. Dimensional stability of the crosslinked compounds will be measured by means of storage tests under different moisture and temperature conditions. Finally, the crosslinking of externally plasticized CA during continuous melt processing in a commercial twin-screw extruder will be investigated.

## References

[1] Schützenberger P. (1865). Action de l'acide acétique anhydre sur la cellulose, l'amidon, les sucres, la mannite et ses congénères, les glucosides et certaines matières colorantes végétales. Compt. Rend. Hebd. Séances Acad. Sci..

[2] Mohanty A.K., Wibowo A., Misra M., Drzal L.T. (2003). Development of renewable resource-based cellulose acetate bioplastic: Effect of process engineering on the performance of cellulosic plastics. Polym. Eng. Sci..

[3] Fridman O.A., Sorokina A.V. (2006). Criteria of efficiency of cellulose acetate plasticization. Polym. Sci. Ser. B.

[4] Fridman O.A. (2013). Structural-relaxation mechanism of glassy-like polymers plasticization. Am. J. Polym. Sci..

[5] Zepnik S., Kabasci S., Kopitzky R., Radusch H.-J., Wodke T. (2013). Extensional flow properties of externally plasticized cellulose acetate: Influence of plasticizer content. Polymers.

[6] Nishio N. (2006). Material functionalization of cellulose and related polysaccharides via diverse microcompositions. Adv. Polym. Sci..

[7] Hamburger C.J. (1967). The effect of chain growth retardation in the graft polymerization of styrene onto cellulose acetate. Ph.D. Thesis.

[8] Vidéki B., Klébert S., Pukánszky B. (2007). External and internal plasticization of cellulose acetate with caprolactone: Structure and properties. J. Polym. Sci. B Polym. Phys..

[9] Sobue H., Matsuzaki K., Komagata H., Ishida A. (1963). Grafting of styrene on cellulose acetate films. J. Polym. Sci. C Polym. Symp..

[10] Biermann C.J., Chung J.B., Narayan R. (1987). Grafting of polystyrene onto cellulose acetate by nucleophilic displacement of mesylate groups using the polystyrylcarboxylate anion. Macromolecules.

[11] Billy M., da Costa A.R., Lochon P., Clément R., Dresch M., Etienne S., Hiver J.M., David L., Jonquières A. (2010). Cellulose acetate graft copolymers with nano-structured architectures: Synthesis and characterization. Eur. Polym. J..

[12] Kostrov Y.A., Litovchenko G.D. (1969). Cross-linking of acetate fibres with blocked diisocyanate. Fibre Chem..

[13] Ghatge N.D., Sabne M.B., Gujar K.B., Mahajan S.S. (1984). Modified cellulose acetate membranes for desalination. J. Appl. Polym. Sci..

[14] Mahajan S.S., Sabne M.B., Gujar K.B., Ghatge N.D. (1985). Selectivity of isocyanate modified cellulose acetate membranes to sugars. Int. J. Polym. Mater..

[15] Stiubianu G., Cazacu M., Nicolescu A., Hamciuc V., Vlad S. (2010). Silicone-modified cellulose. Crosslinking of the cellulose acetate with 1,1,3,3-tetramethyldisiloxane by Pt-catalyzed dehydrogenative coupling. J. Polym. Res..

[16] Nie L., Narayan R. (2003). Grafting cellulose acetate with styrene maleic anhydride random copolymers for improved dimensional stability of cellulose acetate. J. Appl. Polym. Sci..

[17] Yoshioka M., Hagiwara N., Shiraishi N. (1999). Thermoplasticization of cellulose acetates by grafting of cyclic esters. Cellulose.

[18] Warth H., Mülhaupt R., Schätzle J. (1997). Thermoplastic cellulose acetate and cellulose acetate compounds prepared by reactive processing. J. Appl. Polym. Sci..

[19] Klébert S. (2007). Modification of Cellulose Acetate by Reactive Processing—Chemistry, Structure and Properties. Ph.D. Thesis.

[20] Klébert S., Nagy L., Domján A., Pukánszky B. (2009). Modification of cellulose acetate with oligomeric polycaprolactone by reactive processing: Efficiency, compatibility, and properties. J. Appl. Polym. Sci..

[21] Lee S.H., Shiraishi N. (2001). Plasticization of cellulose diacetate by reaction with maleic anhydride, glycerol, and citrate esters during melt processing. J. Appl. Polym. Sci..

[22] Teramoto Y., Yoshioka M., Shiraishi N., Nishio Y. (2002). Plasticization of cellulose diacetate by graft copolymerization of ε-caprolactone and lactic acid. J. Appl. Polym. Sci..

[23] Redl A., Morel M.H., Bonicel J., Guilbert S., Vergnes B. (1999). Rheological properties of gluten plasticized with glycerol: Dependence on temperature, glycerol content and mixing conditions. Rheol. Acta.

[24] Vera-Graziano R., Hernandez-Sanchez F., Cauich-Rodriguez J.V. (1995). Study of crosslinking density in polydimethylsiloxane networks by DSC. J. Appl. Polym. Sci..

[25] (2014). Kunststoffe–Thermoplaste–Bestimmung der Vicat-Erweichungstemperatur (VST).

[26] Gao Z.H., Gu J.Y., Wang X.-M., Li Z.G., Bai X.D. (2005). FTIR and XPS study of the reaction of phenyl isocyanate and cellulose with different moisture contents. Pigment Resin Technol..

[27] Cailloux J., Santana O.O., Franco-Urquiza E., Bou J.J., Carrasco F., Gámez-Pérez J., Maspoch M.L. (2013). Sheets of branched poly(lactic acid) obtained by one step reactive extrusion calendering process: Melt rheology analysis. Express Polym. Lett..

[28] Hsieh K.H., Lin B.Y., Chiu W.Y. (1989). Studies on diisocyanate-modified cellulose acetate membranes. Desalination.

[29] Botaro V.R., Gandini A. (1998). Homogeneous chemical modification of cellulose acetate using different isocyanates. Polímeros.

[30] Xing Q., Yates K., Vogt C., Qian Z., Frost M.C., Zhao F. (2014). Increasing mechanical strength of gelatin hydrogels by divalent metal ion removal. Sci. Rep..

[31] Mezger T.G. (2006). Das Rheologie Handbuch: Für Anwender von Rotations-und Oszillations-Rheometern.

[32] Chetty A., Kovács J., Sulyok Zs., Mészáros Á., Fekete J., Domján A., Szilágyi A., Vargha V. (2013). A versatile characterization of poly(*N*-isopropylacrylamide-*co*-*N*,*N*'-methylene-bis-acryl-amide) hydrogels for composition, mechanical strength, and rheology. Express Polym. Lett..

[33] Winter H.H., Mours M. (1997). Rheology of polymers near liquid–solid transitions. Adv. Polym. Sci..

[34] Wolff F., Kugler C., Münstedt H. (2011). Viscoelastic properties of a silicone resin during crosslinking. Rheol. Acta.

[35] Niamlang S., Sirivat A. (2008). Electromechanical responses of a crosslinked polydimethylsiloxane. Macromol. Symp..

[36] Broedersz C.P., Kasza K.E., Jawerth L.M., Münster S., Weitz D.A., MacKintosh F.C. (2010). Measurement of nonlinear rheology of cross-linked biopolymer gels. Soft Matter.

[37] Levita G., de Petris S., Marchetti A., Lazzeri A. (1991). Crosslink density and fracture toughness of epoxy resins. J. Mater. Sci..

[38] Lange J., Luisier A., Hult A. (1997). Influence of crosslink density, glass transition temperature and addition of pigment and wax on the scratch resistance of an epoxy coating. J. Coating Technol..

[39] Hale A., Macosko C.W., Bair H.E. (1991). Glas transition temperature as a function of conversion in thermosetting polymers. Macromolecules.

[40] Chang T.D., Carr S.H., Brittain J.O. (1982). Studies of epoxy resin systems: Part B: Effect of crosslinking on the physical properties of an epoxy resin. Polym. Eng. Sci..

[41] Reddy N., Yang Y. (2010). Citric acid cross-linking of starch films. Food Chem..

[42] Ambrogi V., Brostow W., Carfagna C., Pannico M., Persico P. (2012). Plasticizer migration from cross-linked flexible PVC: Effects on tribology and hardness. Polym. Eng. Sci..

[43] Lakshmi S., Jayakrishnan A. (1998). Photocross-linking of dithiocarbamate-substituted PVC reduces plasticizer migration. Polymer.

